# A germanium hole spin qubit

**DOI:** 10.1038/s41467-018-06418-4

**Published:** 2018-09-25

**Authors:** Hannes Watzinger, Josip Kukučka, Lada Vukušić, Fei Gao, Ting Wang, Friedrich Schäffler, Jian-Jun Zhang, Georgios Katsaros

**Affiliations:** 1 Institute of Science and Technology Austria, Am Campus 1, 3400 Klosterneuburg, Austria; 2 National Laboratory for Condensed Matter Physics and Institute of Physics, Chinese Academy of Sciences, 100190 Beijing, China; 3 Johannes Kepler University, Institute of Semiconductor and Solid State Physics, Altenbergerstr, 69, 4040 Linz, Austria; 4 CAS Center for Excellence in Topological Quantum Computation, University of Chinese Academy of Sciences, 100190 Beijing, China

## Abstract

Holes confined in quantum dots have gained considerable interest in the past few years due to their potential as spin qubits. Here we demonstrate two-axis control of a spin 3/2 qubit in natural Ge. The qubit is formed in a hut wire double quantum dot device. The Pauli spin blockade principle allowed us to demonstrate electric dipole spin resonance by applying a radio frequency electric field to one of the electrodes defining the double quantum dot. Coherent hole spin oscillations with Rabi frequencies reaching 140 MHz are demonstrated and dephasing times of 130 ns are measured. The reported results emphasize the potential of Ge as a platform for fast and electrically tunable hole spin qubit devices.

## Abstract (web summary)

Germanium’s electronic structure and large, tunable spin-orbit coupling makes it a good material for constructing hole-based quantum devices. Here the authors demonstrate the fabrication and two-axis control of a hole spin qubit in a germanium double quantum dot.

## Introduction

Spins in isotopically purified Si have shown record coherence times^1^ and fidelities^2^ making them promising candidates for scalable quantum circuits^3^. One of the key ingredients for realizing such circuits will be a strong coupling of spins to superconducting resonators^4^. This has been recently achieved for Si by dressing electrons with synthetic spin-orbit coupling^5,6^. Ge, on the other hand, with its strong and tunable spin-orbit coupling^7–10^ could be an alternative material for the realization of scalable qubits.

In the past few years several studies have addressed the properties of Ge/Si core/shell nanowires and Ge self-assembled nanocrystals^8,11–13^. Here, we study hut wires (HWs), Ge nanowires monolithically grown on Si. They have a triangular cross section with a width of about 20 nm and a height of about 2 nm^14–18^. As has been very recently reported^16^, holes localized in Ge HWs are of almost pure heavy-hole (HH) character making them thus an appealing system for hosting hole qubits with long dephasing times^19^.

In this work we demonstrate the ability to capture holes in double quantum dots (DQDs) fabricated from Ge HWs. We make use of the Pauli spin blockade (PSB)^20^ mechanism and the electric dipole spin resonance (EDSR) technique in order to demonstrate the addressability of single holes. By varying the duration of the radio frequency (RF) burst, Rabi oscillations with frequencies higher than 100 MHz are observed. Finally, Ramsey fringes-like measurements reveal dephasing times of 130 ns, twice the dephasing time reported for holes in Si^21^.

## Results

### Double quantum dot and Pauli spin blockade

**Fig. 1:**
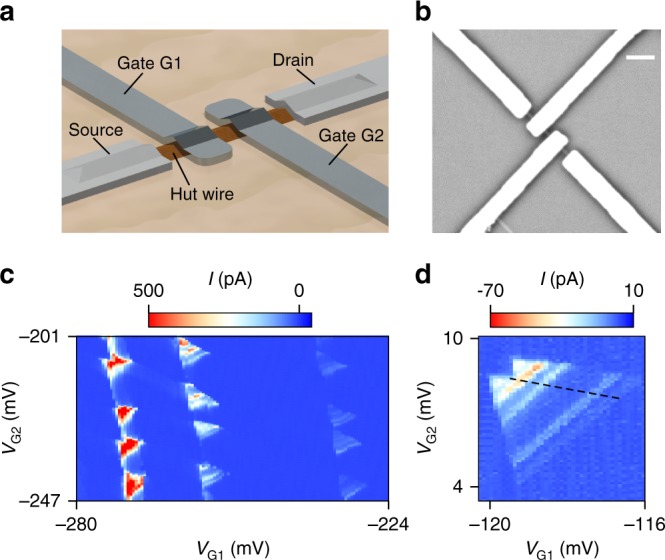
DQD devices from Ge HWs. **a** Schematic showing a Ge HW contacted with source and drain electrodes and covered by two top gates (G1, G2). The hafnium oxide layer separating the HW from the gates is not shown. **b** Scanning electron micrograph of a device similar to the ones measured. The scale bar is 200 nm. **c** Stability diagram of DQD device A showing the characteristic bias triangles at a bias voltage of *V*_SD_ = 2 mV. **d** Representative zoom-in of a pair of bias triangles at *V*_SD_ = −2 mV from device B. The dashed black line indicates the edge of the lower bias triangle

A schematic and a scanning electron micrograph of a typical DQD device are shown in Fig. 1a, b, respectively. In a first step, we probe our DQD by applying a source-drain voltage V_SD_ and measuring the resulting current *I* to test our DQD device. Thereby, we vary the voltages *V*_G1_ and *V*_G2_ tuning the electrochemical potentials of our dots. The low temperature measurements reveal the formation of quantum dots (QDs) below the deposited top gates, presumably due to strain^22^. Therefore, the two gates (with voltages *V*_G1_ and *V*_G2_) are already sufficient to fully define and operate the DQD. The stability diagram of the DQD device A showing characteristic bias triangles^23^ is depicted in Fig. 1c. For comparison, a representative measurement of two bias triangles from the second device B is shown in Fig. 1d. Due to the fairly low mutual capacitance^24^ of about 1 aF the triangles are merged already at relatively low bias voltages. The base of the triangle marks current flowing through the ground states. The parallel lines within the triangles denote transport through excited states. Energy level separations of up to ~1 meV and a relative lever arm Δ*V*_G1_/Δ*V*_G2_ = 0.7 are observed^23^. Since the two top gates G1 and G2 are very close to the HW, a relatively strong coupling is obtained, leading to large gate couplings of *α*_1_ = 0.62 eVV^−1^ and *α*_2_ = 0.43 eVV^−1^^24^.

**Fig. 2:**
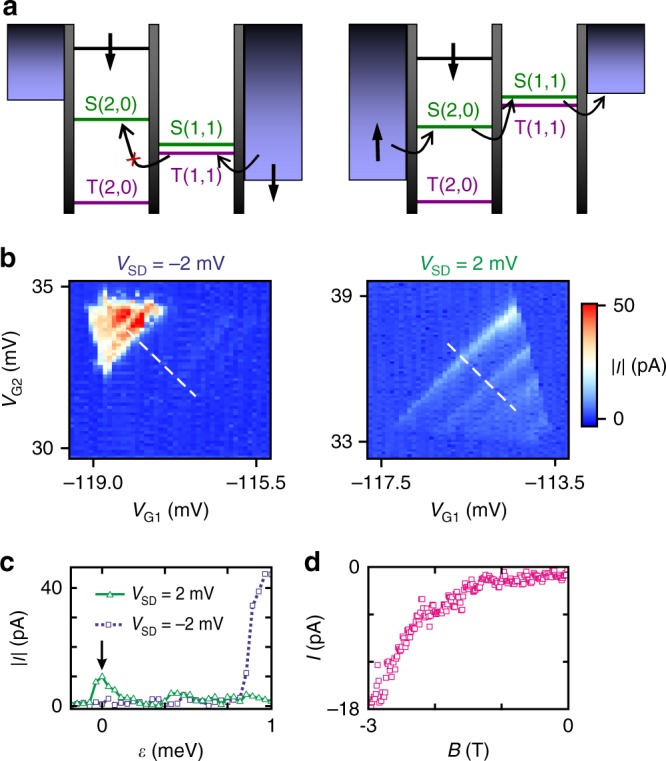
Spin blockade in DQDs. **a** Schematic presentation of PSB for a hole DQD. Transport is blocked for the transition (1, 1) → (2, 0) (left panel) due to the Pauli exclusion principle and can be lifted by reversing the applied bias voltage (right panel). **b** Bias triangles exhibiting PSB at negative bias voltages (left). Reversing the bias results in an enhancement of the baseline current (right). **c** Comparison of the current from two line cuts along the detuning axis for positive (green triangles, solid line) and negative (blue squares, dotted line) bias voltages. The positions where the line cuts were taken are indicated by white dashed lines in **b**. **d** Dependence of the leakage current at zero detuning for an out-of-plane magnetic field and for a bias voltage of −2 mV

In order to realize a spin 3/2 qubit in the DQD devices we rely on PSB as a spin-selective read-out mechanism^25,26^. PSB occurs in a (1, 1) → (2, 0) or an equivalent (2N−1, 2N−1) → (2 N, 2N−2) charge configuration (Fig. 2a), where the first (second) number is the number of holes in the left (right) dot, respectively. In such a configuration transport through the DQD is blocked since the triplet (2, 0) state is lying too high in energy. Reversing the applied source-drain voltage lifts the blockade. Signatures of PSB were observed in several bias triangles exhibiting a suppressed leakage current of the triangle baseline. Two representative direct current measurements are shown in the left and right panel of Fig. 2b for bias voltages of −2 mV and + 2 mV, respectively. The corresponding line traces along the detuning direction (white dashed lines) are plotted below in Fig. 2c. In the blocked configuration (blue squares, dotted line) the zero-detuning current, indicated by the black arrow, drops to about 2 pA compared to 10 pA in the non-blocked case (green triangles, solid line), as expected for PSB^23^. The magnetic field dependence of the leakage current in the blocked configuration is shown in Fig. 2d for an out-of-plane magnetic field. The clear increase of the leakage current at elevated magnetic fields is an indication for a spin-orbit induced lifting mechanism of PSB^27,28^, though this is expected already at much lower magnetic fields. At zero magnetic field, no nuclear-spin induced current peak can be observed^23^, which indicates a low hyperfine interaction.

### Electric dipole spin resonance

**Fig. 3:**
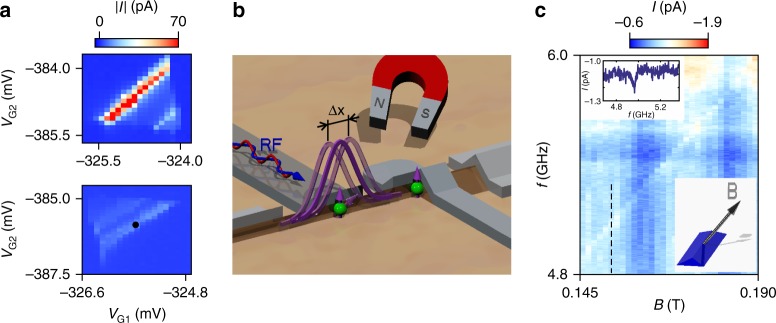
EDSR spectroscopy. **a** Set of bias triangles from device C for *V*_SD_ = 1 mV (upper panel) and *V*_SD_ = −1 mV (lower panel). **b** Illustration showing the principle of EDSR. An RF signal applied to the top gate can change the position of the hole wave function. Such an oscillating motion in combination with a static magnetic field can lead to spin rotations for a system with strong spin-orbit coupling. **c** Raw data measurement showing the frequency versus magnetic field dependence of the zero detuning current measured at the position marked by the black circle in **a**. For a reversed bias voltage no such EDSR response could be observed (Supplementary Fig. 2). The magnetic field is oriented 45° in respect to both the out-of-plane direction and the in-plane HW axes (see lower inset). The upper inset shows a line trace taken along the black dashed line

We now add an RF electric field on top of the static voltage applied to one of the two top gates. Such can rotate one of the spins and thus lift PSB. This is achieved via the EDSR mechanism^29^. An RF electric field applied to one of the two gates of the DQD (here G1) can cause oscillations in the position of the confined hole wave function (Fig. 3b). Such an oscillation in combination with a constant applied magnetic field leads to spin rotations in systems with strong spin-orbit coupling^23^. In order to induce such continuous wave spin rotations the driving frequency of the RF electric field has to be equal to the Larmor frequency *f*_0_ = |*g*|*μ*_B_*B*/*h*, where *g* is the *g*-factor for a certain magnetic field orientation, *μ*_B_ is the Bohr magneton and *h* is Planck’s constant.

Figure 3a shows a pair of bias triangles for positive and negative bias voltages from the third measured device C. The hole number in each dot is estimated to be about 11 (Supplementary Fig. 1). Compared to device B the width of the gates for this device was increased from about 60 nm to about 120 nm in order to reduce the spatial confinement of the hole wave function and therefore increase the EDSR response. The black circle in the lower panel of Fig. 3a indicates the position at which the EDSR measurement shown in Fig. 3c was performed. From the slope of the resonance line a *g*-factor of ~2 can be extracted.

**Fig. 4:**
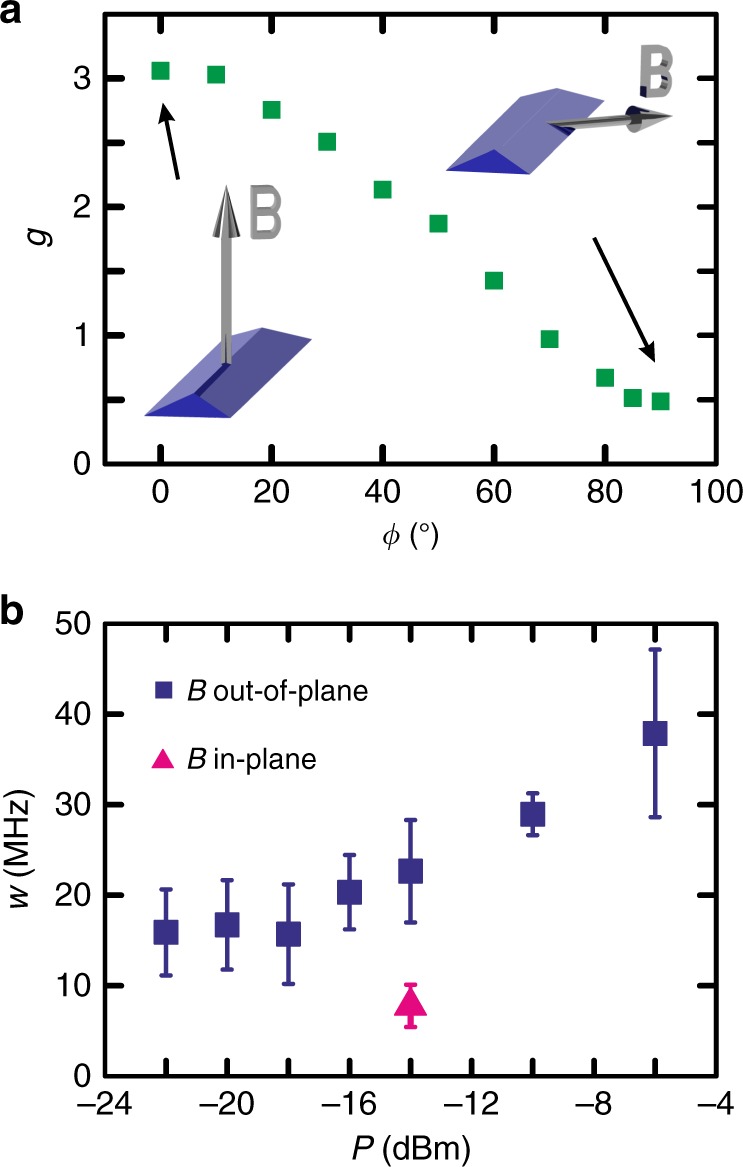
Angle dependence of the *g*-factor and power dependence of the EDSR peak width. **a** Extracted *g*-factor values for different magnetic field orientations as illustrated by the two insets. *ϕ* denotes the angle of the magnetic field vector **B** to the [001] direction; i.e., *ϕ* = 0° (90°) corresponds to an out-of-plane (in-plane) magnetic field. The *g*-factors were extracted from a linear fit to the values obtained by a Gaussian fit to the EDSR peaks at several positions. The s.d. errors are below 0.03 for all data points and therefore not visible. The measurements were taken with an RF power of −14 dBm. **b** Power dependence of the FWHM *w* of the resonance peak from Fig. 3b for an out-of-plane magnetic field (blue squares). For examples of the line traces see Supplementary Fig. 5. Below −16 dBm the width saturates at ~16 MHz. However, for an in-plane magnetic field and for a power of −14 dBm the EDSR peak width shrinks to 7.8 ± 2.3 MHz (red triangle). For lower power values the EDSR line is not any more visible. The s.e.m. error bars were extracted from averaging over all values obtained from Gaussian fits to the resonance peaks at the respective power

By changing the direction of the magnetic field the slope of the EDSR line is changing due to the direction dependence of the *g*-factor. Each of the *g*-factor values shown in Fig. 4a was extracted from a linear fit through several points along the respective resonance line. The *g*-factor values show a strong anisotropy in good agreement with earlier experimental findings for HH states^16^.

### Dependence of $T_2^ \ast$ on the magnetic field direction

EDSR does not only lift PSB, but also allows the extraction of a lower limit for the hole spin dephasing time $T_2^ \ast$. In order to extract this lower bound for $T_2^ \ast$, the power *P* of the applied RF signal was varied. At high power, the EDSR width is power broadened (see also Supplementary Note 3). However, for measurements taken in an out-of-plane magnetic field the width is saturating at values of about −18 dBm, as can be seen in Fig. 4b. Therefore, a lower bound for the dephasing time of ~33 ns can be extracted using the relation $T_2^ \ast = 2\sqrt {{\mathrm{ln}}(2)} /(\pi \omega )$, where *w* is the full width at half maximum (FWHM) of the resonance peak at a certain RF power^30^. For HH states it has been predicted that the direction of the applied magnetic field has a strong influence on the dephasing times^19^. Indeed, optical measurements of hole spins confined in GaAs self-assembled QDs have shown very long dephasing times^31^. In order to obtain such longer dephasing times, the external magnetic field needs to be aligned perpendicular to the direction of the Overhauser field, which for HH states is perpendicular to the growth plane^19^. By repeating the EDSR measurement for an in-plane magnetic field and an RF power of −14 dBm (see Supplementary Fig. 4), we obtain a lower bound of 68 ns for the dephasing time.

### Coherent spin oscillations and two-axis control

**Fig. 5:**
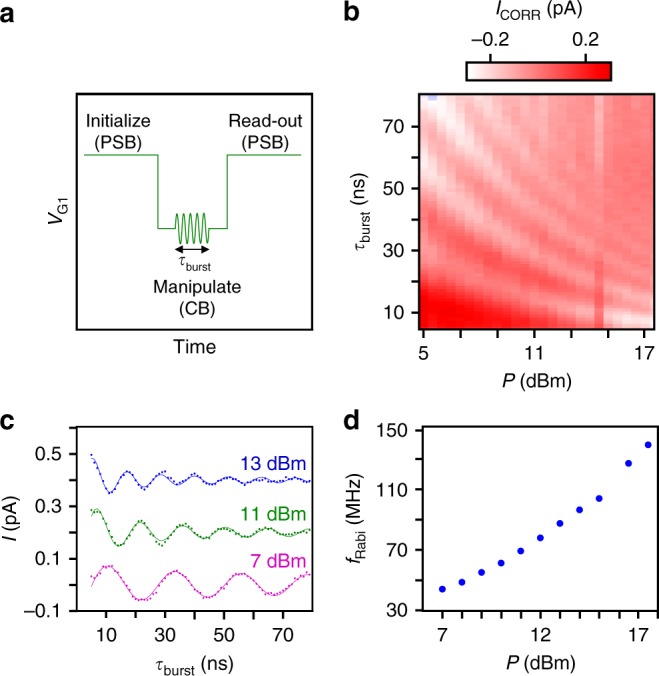
Coherent spin oscillations. **a** Voltage signal applied to G1 for performing the Rabi experiment. The square pulse shifts the qubit between PSB and Coulomb blockade (CB), lasting for 210 and 100 ns, respectively. The applied RF burst rotates the initialized spin-down vector for an angle proportional to its duration *τ*_burst_ and the square root of power $\sqrt P$. **b** Corrected current *I*_CORR_ versus *τ*_burst_ and *P* for an out-of-plane magnetic field of 127 mT and an RF frequency of 5.966 GHz for device C after thermal cycling, showing Rabi oscillations. The current data has been corrected by removing the average column and row values from the corresponding data points. **c** Current *I*_CORR_ vs *τ*_burst_ traces for *P* = 7, 11 and 13 dBm. For clarity, the traces for 11 and 13 dBm are shifted by 200 and 400 fA, respectively. The Rabi frequencies were extracted by fitting the raw data to $\frac{A}{{\tau _{{\mathrm{burst}}}^a}}\,{\mathrm{sin}}\,(2\pi f_{{\mathrm{Rabi}}}\tau _{{\mathrm{burst}}} + \phi ) + {\mathrm{offset}} + c\sqrt {\tau _{{\mathrm{burst}}}}$. The offset and $c\sqrt {\tau _{{\mathrm{burst}}}}$ have been subtracted from the shown traces. **d** Dependence of the Rabi frequency on the applied RF power

In order to demonstrate coherent control over the hole spin state, a voltage signal is applied to G1 as can be seen in Fig. 5a. The system is initialized in the triplet state. When in CB, an RF burst of varying duration is applied. For a π-pulse the hole spin will flip leading thus to a singlet (1, 1) state. The system is then brought back into the PSB region for spin read-out and the hole can tunnel to the singlet (2, 0) state leading to an enhanced current. By linearly increasing the duration of the RF burst, oscillations of the detected current can be observed (Fig. 5b). As expected, the period of the Rabi oscillations decreases with increasing power of the RF burst (Fig. 5c). Rabi frequencies approaching 140 MHz are observed (Fig. 5d). They are faster than what has been predicted for Ge nanocrystals^32^ and than those reported for the InSb electron spin qubit which showed 8 ns dephasing time^33^.

**Fig. 6:**
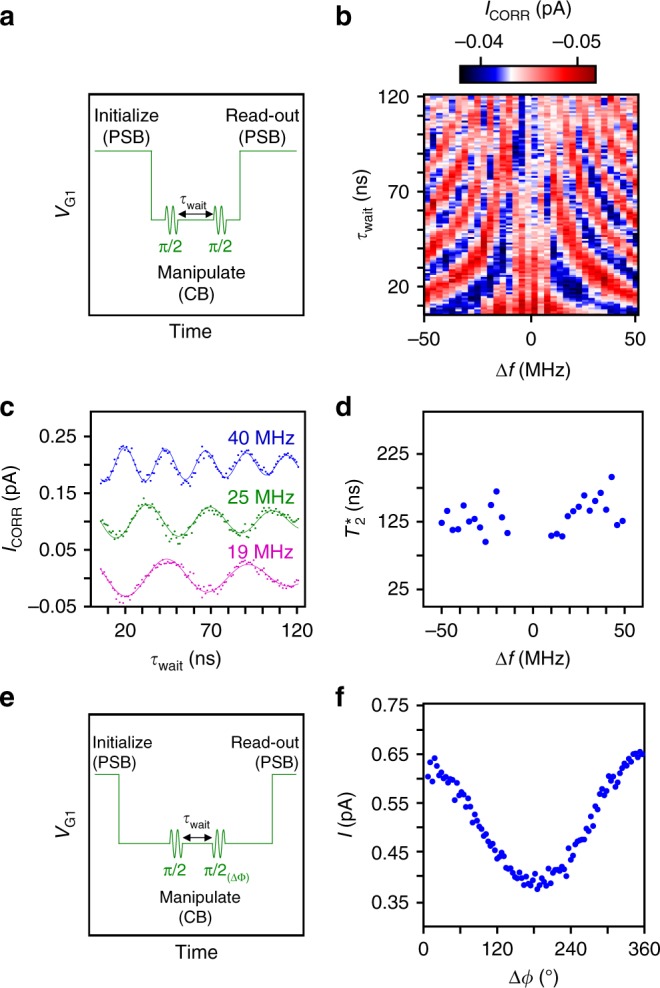
Two-axis qubit control. **a** Voltage signal applied to G1 for inducing Ramsey oscillations caused by the frequency detuning Δ*f* between the Larmor and the RF burst frequency. The square pulse shifts the qubit between PSB (210 ns) and CB (160 ns). Each of the two RF bursts having the same phase are separated by the waiting time *τ*_wait_ and rotate the spin vector for a ${\textstyle{\pi \over 2}}$ angle. **b** *I*_CORR_ versus *τ*_wait_ and Δ*f* for *P* = 11 dBm and *τ*_burst_ = 3.5 ns in presence of an out-of-plane magnetic field of 127 mT and at a center frequency of 5.966 GHz. **c** Line traces from **b** for Δ*f* =19, 25 and 40 MHz. The traces for 25 and 40 MHz are shifted by 100 and 200 fA, respectively. **d** Plot showing the extracted dephasing times for various values of Δ*f*. The times were extracted by fitting the data to *A*$e^{ - ( {\frac{{\tau _{{\mathrm{wait}}}}}{{T_2^ \ast }}} )^2}$sin (2π Δ*fτ*_wait_ + *ϕ*) + offset. The average dephasing time exceeds 130 ns. **e** Voltage signal applied to G1 for performing a two-axis qubit rotation caused by Δ*ϕ* between the two RF bursts . The manipulation interval lasted for 160 ns, while the read-out and the initialization lasted for 30 ns each. **f** Current passing through the DQD versus Δ*ϕ*. The oscillatory behavior of the current demonstrates the qubit rotation around the axis defined by Δ*ϕ*. For this experiment the following parameters were chosen: *P* = 4 dBm, *τ*_burst_ = 9 ns, *τ*_wait_ = 10 ns, *f* = 5.887 GHz and an out-of-plane magnetic field of 122 mT

To measure the inhomogeneous dephasing time $T_2^ \ast$, a Ramsey experiment was performed. A periodic voltage signal was applied to G1 as shown in Fig. 6a. Two $\frac{\pi }{2}$-pulses, separated by *τ*_wait_ during which the qubit can freely evolve and dephase, were applied during the manipulation interval. For each value Δ*f* the current oscillates as a function of *τ*_wait_ (Fig. 6b). From the decay time of these oscillations, average dephasing times exceeding 130 ns were measured (Fig. 6c, d). The ratio of $T_2^ \ast$ to *τ*_π_ for an RF power of 11 dBm is ~18 which is 35 times smaller than the highest value reported for electron spins in isotopically purified ${\rm {Si}}^2$, but just a factor of two compared to electron spins in natural Si^34^. Due to the limited visibility in our experiment caused by the small current flowing through the DQD, it was not possible to extend *τ*_wait_ further than 160 ns. This prohibited the investigation of longer $T_2^ \ast$, possibly arising for parallel magnetic fields as shown in Fig. 4b.

In order to demonstrate an alternative two-axis qubit rotation, a similar pulse scheme as before was applied, but for this experiment the phase difference Δ*ϕ* between the two $\frac{\pi }{2}$-pulses was swept (Fig. 6e). This phase difference defined the second rotation axis. Sweeping Δ*ϕ* linearly from 0 to 360^*o*^ causes a sinusoidal oscillation of the projected spin-up fraction and consequently of the measured current through the DQD (Fig. 6f).

## Discussion

While the obtained results are a first step towards fast hole spin qubits with longer dephasing times, the measured $T_2^ \ast$ times are still three to four times lower than those extracted from optical measurements for hole spins in self-assembled InGaAs QDs^31^. Future experiments will focus on the effect of charge noise and how to radically reduce it. By moving then to isotopically purified Si and Ge, qubits with long coherence times, limited just by the spin relaxation time^35^ should be feasible.

In conclusion, by using PSB in a DQD device we have demonstrated a Ge hole spin qubit allowing arbitrary rotations around two axes. Despite the strong spin-orbit coupling, the obtained $T_2^ \ast$ is higher than that of holes^21^ confined in QDs formed in natural Si and just one order of magnitude lower than that of electrons^34^. The reported results combined with the possibility of self-organization^36,37^ pave the way towards more complex hole qubit devices.

## Methods

### Device fabrication

The Ge HWs characterized in this work were grown by solid-source molecular beam epitaxy (MBE) on 4-inch intrinsic Si(001) wafers in two different systems. Two different wafers were used for the realization of the three devices A, B and C, which differ in several growth parameters, as can be seen in Table 1. The wafers were dipped in an HF solution before loading into the MBE chamber. After degassing at 720 °C, a Si buffer layer was deposited. Then 6.7 Å (6.5 Å) of Ge were deposited on the substrate at 580 °C (545 °C) followed by an in-situ annealing of 5 h (4 h) at 570 °C (535 °C) for device A and C (device B). The amount of the deposited Ge is at the critical thickness for the nucleation of three dimensional hut clusters. At last, the substrate temperature was decreased to 300 °C and capped with 5 nm (3 nm) Si for device A and C (device B).

**Table 1:** Growth and fabrication parameters

	Device A	Device B	Device C
Deposited Ge (Å)	6.7	6.5	6.7
Growth temp. (°C)	580	545	580
Annealing temp. (°C)	570	535	570
Annealing time (h)	5	4	5
Si cap thickness (nm)	5	3	5
Source/drain (nm)	Pt 25	Pd/Al 5/25	Pt 25
Gates (nm)	Ti/Pt 3/25	Ti/Pt 3/25	Ti/Pt 3/25

Parameters used for the growth and the fabrication of devices A, B and C

Device A and C (device B) were fabricated using a 100 kV (20 kV) e-beam lithography system. For the source and drain contacts 25 nm Pt (5/25 nm Pd/Al) were deposited. The gates (3/25 nm Ti/Pt) were evaporated onto an about 6–8 nm hafnium oxide layer. The oxide was created by atomic layer deposition of 80 cycles of Tetrakis(dimethylamido)hafnium (Tetrakis(ethylmethylamido)hafnium)/80 cycles of water at 130 °C (150 °C).

### Experimental setup

All the measurements were done with low-noise electronics and in a He-3/He-4 dilution refrigerator at a base temperature of ~40 mK. A current to voltage amplifier with a gain of 10^9^ was used for the current measurements. All low-frequency lines are filtered at three stages. Pi filters are used at room temperature, LC filters at the mixing chamber stage and a single stage RC filters on the printed circuit board (PCB) on which the sample was mounted. High-frequency signals were applied to the gate G1 through a 20 GHz bandwidth coaxial line and attenuated by 44 dB from attenuators distributed at the different stages of the dilution refrigerator. To apply periodic square voltage pulses for fast switching between the CB and the PSB regime, one channel of an arbitrary wave generator Tektronix AWG5014C was used. Two other channels were connected to the I and Q inputs of the R&S SMW200A vector signal generator for the RF burst creation. Both the RF and the voltage square pulses were merged by a diplexer before entering the dilution refrigerator. Such a signal was further merged with a DC signal via a bias tee positioned on the PCB.

## Electronic supplementary material

Supplementary Information

## Data Availability

The data that support the findings of this study are available from the corresponding author upon reasonable request.
