# Supplementary material for: A germanium hole spin qubit
Source: Nat Commun. 2018 Sep 25;9:3902. doi: 10.1038/s41467-018-06418-4 (PMC6156604; doi:10.1038/s41467-018-06418-4)
Supplement: Supplementary file 1 — Supplementary Information [file 41467_2018_6418_MOESM1_ESM.pdf]

Supplementary Information: A germanium hole spin qubit

Hannes Watzinger, Josip Kukučka et al.

Supplementary Note 1: Number of confined holes in device C
The number of confined holes in the DQD can be extracted from its stability diagram. Supplementary Figures 1(a) and (b) show DC measurements at $V_{\mathrm{SD}} = 5\,\mathrm{mV}$ of V_{G2} versus V_{G1} and V_{G1} versus V_{G2}, respectively. From the point where the two gates are switching the dots off to the triangles highlighted by the white dashed square one can estimate a hole number of about 11 in each dot.

Supplementary Note 2: EDSR measurements for both bias directions
Supplementary Figure 2(a) shows the same EDSR measurement as in Fig. 3 in the main manuscript with the background subtracted. For comparison, an EDSR measurement for the reversed bias voltage is presented in Supplementary Figure 2(b) showing no EDSR response at a similar frequency and magnetic field range. Both measurements were performed using the same RF power and at the base line of the opposite bias triangles.

Supplementary Note 3: Actual voltage arriving at the device
In order to extract the actual power from the applied RF signal reaching our device, the power dependent broadening of the triangle base line was investigated. Supplementary Figure 3 shows bias triangles without an applied RF signal in (a) and with an applied RF wave of -20 dBm and -15 dBm in (b) and (c), respectively.

Supplementary Note 4: EDSR measurement for an in-plane magnetic field
The measurement, from which the FWHM value of the EDSR peak for an in-plane magnetic field was extracted [see Fig. 4(b) from the main manuscript], is shown in Supplementary Figure 4(a) with a corresponding line trace as an inset. For a power lower than -14 dBm the resonance line could not be properly resolved.

Supplementary Note 5: Power dependence of the resonance line width
Supplementary Figure 5 shows representative line traces of EDSR measurements, which were used to obtain the power dependence of the peak width in Fig. 4(b), for an out-of-plane magnetic field and different RF power. For extracting the peak width, a Gaussian fit was applied to the data.

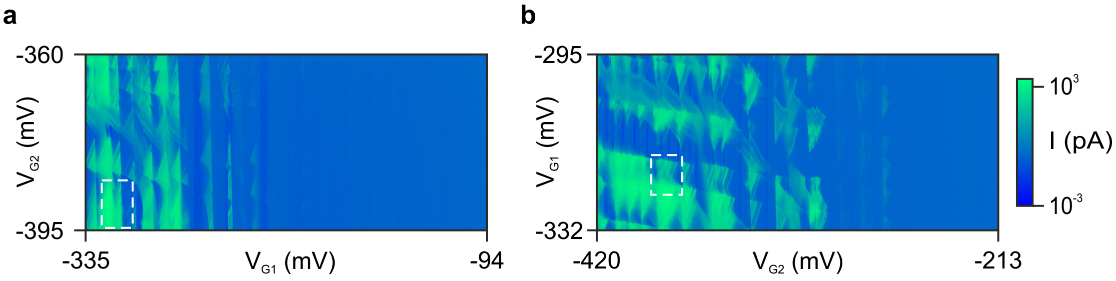

Supplementary Figure 1: **DQD stability diagrams for estimating the number of confined holes.** DC measurements showing the turn-off behavior along V_{G1} (a) and V_{G2} (b) at $V_{SD} = 5\,\text{mV}$. Similar measurements were performed at a higher bias voltage of $V_{SD} = 20\,\text{mV}$, which did not reveal any further triangles at more positive gate voltages. Due to reasons of visibility (merging of the bias triangles) just the measurements with a reduced bias voltage are shown. The dashed white rectangles indicate the triangles from the EDSR measurement in Fig. 3 and 4.

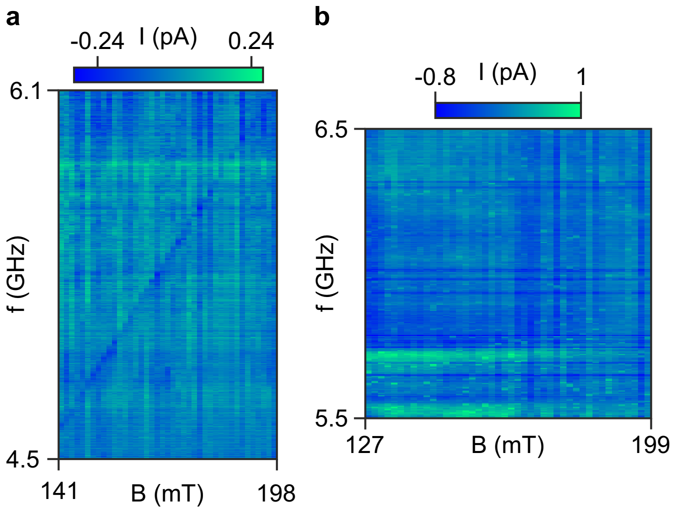

Supplementary Figure 2: **EDSR measurements for opposite bias directions.** Measurements showing the frequency versus magnetic field dependence of the zero detuning current measured at the base line of the bias triangles for negative (a) and positive (b) bias. As expected, just for the negative bias direction, showing PSB, an EDSR peak can be observed. A magnetic field offset of about 30 mT is caused by the hysteretic behavior of the magnet. For the background correction a single line trace was subtracted from the data vertically and horizontally.

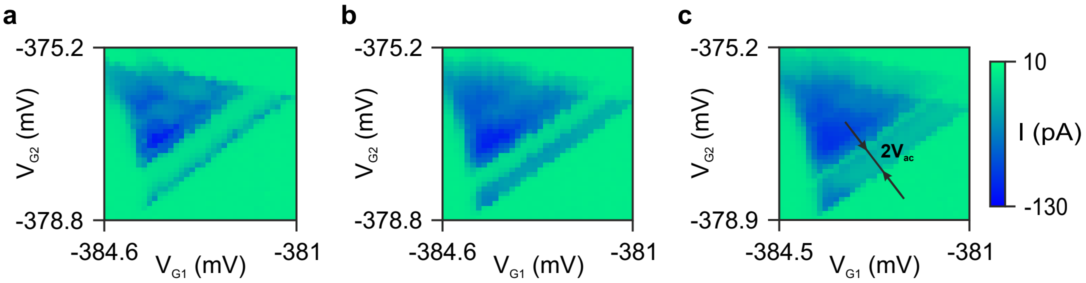

Supplementary Figure 3: **RF power dependence of the bias triangles.** (a) DC measurement of bias triangles with no RF signal applied. (b) and (c) show the same bias triangles with an applied microwave power of -20 dBm and -15 dBm, respectively. From the broadening of the base lines an effective gate modulation $2V_{\mathrm{ac}}$ of 0.75 mV at -20 dBm and of 1.15 mV at -15 dBm can be extracted.

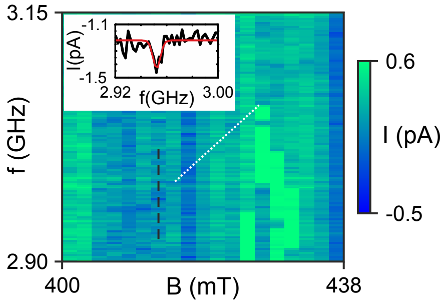

Supplementary Figure 4: **EDSR measurement for an in-plane magnetic field.** Same frequency and magnetic field dependence as shown in Fig. 3, but for an in-plane magnetic field. The white dotted line is a guide to the eye along the resonance line. From the slope of the EDSR line a g-factor of about 0.5 was extracted. The inset shows a line trace taken along the black dashed line together with a Gaussian fit (red) to the EDSR peak.

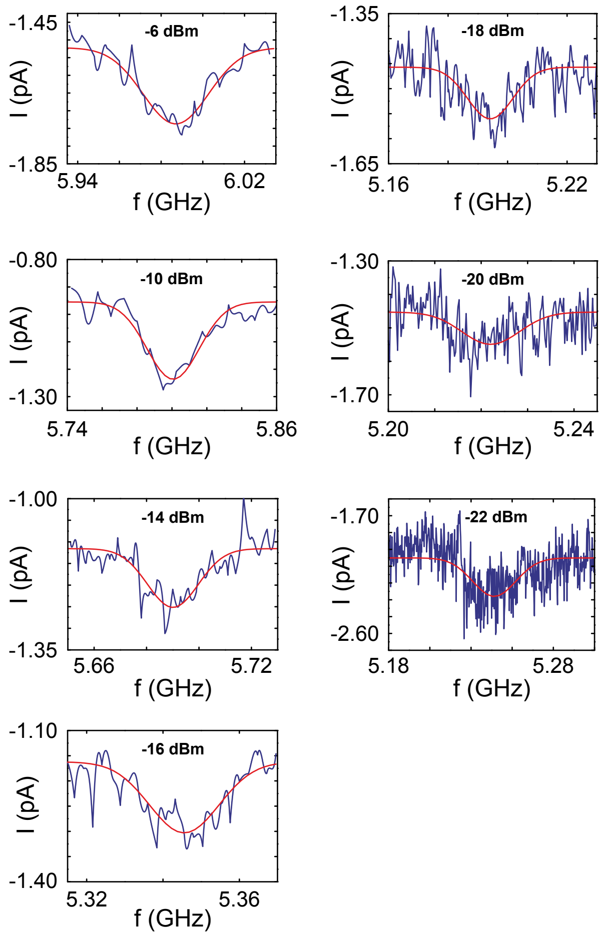

Supplementary Figure 5: **Power dependence of the resonance line width.** Representative line traces showing resonance peaks (blue) for different RF power with the corresponding Gaussian fits (red).
